# Dual-Input Neural Networks for Personalized Image Precompensation

**DOI:** 10.3390/jimaging12090456

**Published:** 2026-09-20

**Authors:** Nafe Alkzir, Maria Yarykina, Alexander Abgaryan, Sergey Gladilin, Dmitry Nikolaev, Ilya Nikolaev

**Affiliations:** 1 Faculty of Computer Science, HSE University, Moscow 109028, Russia; 2 Federal Research Center “Computer Science and Control” of the Russian Academy of Sciences, Moscow 119333, Russialexabgarian@gmail.com (A.A.); gladilin@gmail.com (S.G.);; 3 Institute for Information Transmission Problems of the Russian Academy of Sciences, Moscow 127051, Russia; i.p.nikolaev@iitp.ru; 4 Smart Engines Service LLC, Moscow 117312, Russia

**Keywords:** image precompensation, refractive errors, visual impairments, constrained optimisation, deep learning, image deconvolution

## Abstract

Image precompensation aimed at correcting refractive errors of the human eye seeks to transform a displayed image so that, after being blurred by the observer’s ocular optics, the image projection on the retina closely matches the original picture. The optimal precompensation varies for different refractive errors, so taking into account the specific refractive distortion introduced by a particular eye is called personalization. Existing personalized precompensation methods rely on deconvolution with built-in constraints or further tone mapping, which either produce artifacts or impose contrast loss. In this paper, we propose an approach for adapting modern two-input neural networks developed for non-blind image deconvolution to the problem of personalized image precompensation. The purpose of two inputs is the capability of independently feeding the neural-network model with the image to process and the personal characteristic function of the observer’s eye. Based on the proposed technique, three neural-network models (USRNet-PC, DWDN-PC, and KerUnc-PC) were developed, the adaptation of which was rather a unified approach than individual transformation of each architecture. These models were then comprehensively compared with other modern personalized precompensation methods on the basis of objective quality metrics, computational performance, and the results of simulation-based human studies. It has been shown that USRNet-PC provides the best subjective quality of perception and the shortest processing time on a GPU, while DWDN-PC demonstrates the highest computational efficiency on x86 and ARM CPUs. Thus, a new neural-network approach is proposed for solving the precompensation problem, which appeared to be superior in quality to previously known methods.

## 1. Introduction

Visual impairments such as myopia (nearsightedness), hyperopia (farsightedness), and/or astigmatism are very common: for instance, myopia alone affects 22.9% of the world’s population [1]. These anomalies of the human eye (called refractive errors) cause changes in the perceived image sharpness, which leads to impaired perception and degraded resolution of details in the observed scene. These negative effects are typically compensated for by lenses or contact lenses, although neither the former nor the latter fully compensate for complex anomalies. Moreover, when using virtual reality (VR) headsets, there may be not enough space for glasses, and their use often causes discomfort and reduces the immersion depth. Contact lenses are free of these drawbacks, but it was recently reported [2] that only 140 million of the 2.5 billion myopic people worldwide wear contact lenses. This is due to various reasons, including discomfort, vision problems, cost, and inconvenience.

VR headsets are rapidly evolving [3,4] and can now feature built-in correction systems that use custom lenses or adjust the position of the lens relative to the screen. However, astigmatism compensation remains a technical challenge [5]. On the other hand, modern headsets are striving to miniaturize down to the form factor of glasses [6], which precludes the integration of additional optical devices. Another example: optical correction of refractive errors can be difficult when using smartwatches, which can be useful during sports or at night when glasses or contact lenses are removed.

**Figure 1:**
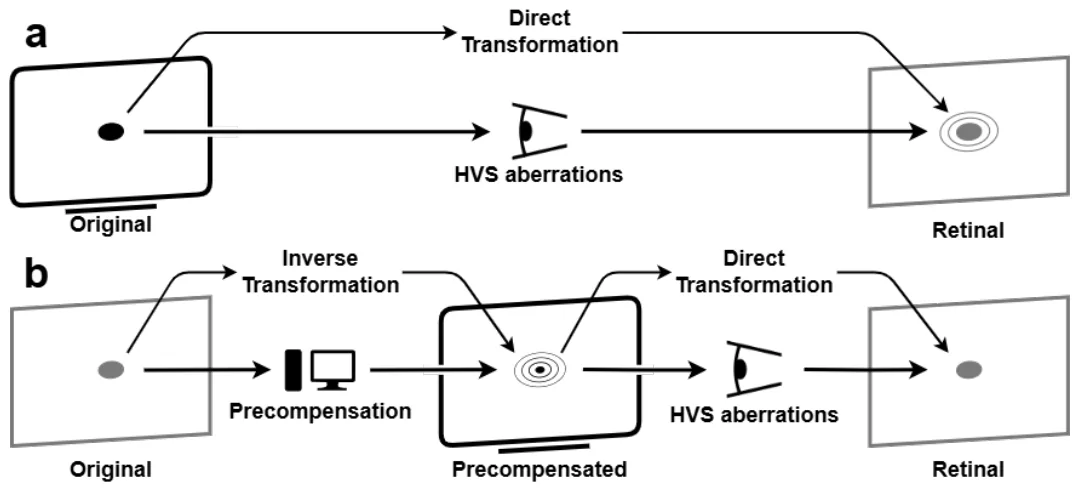
The concept of image precompensation for people with refractive errors of vision: (**a**) direct image formation without precompensation; (**b**) image formation with precompensation. Here, HVS refers to human vision system.

While optical correction methods deal with the observer’s eye, aiming at restoring its optical power to the “proper value”, there is another option to improve the perceived image. This approach, referred to as precompensation, implies embedding a specialized image transform into the device that displays the image to the observer. In this case, the displayed image is being transformed in such a way that, as a result of viewing by the human eye with refractive anomalies, an image (called a *retinal* image) close to the target one is formed on the eye retina (Figure 1). Of course, with this approach, only the perception of the displayed image is improved, and not of the entire environment as a whole.

**Figure 2:**
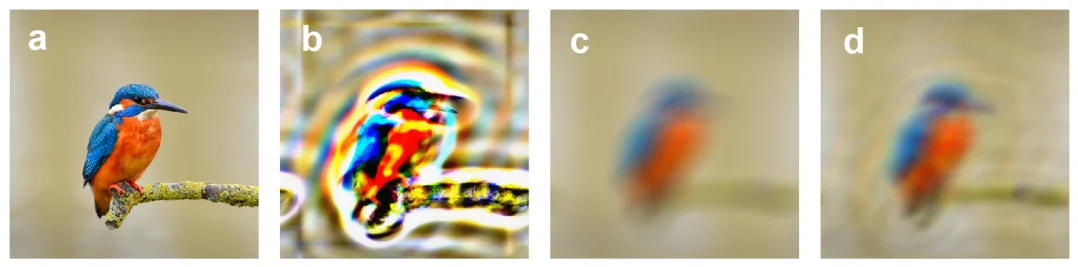
An example of image precompensation: (**a**) original image; (**b**) precompensated image; (**c**) original image on the retina of a myopic eye (simulation); (**d**) pre-compensated image on the retina of a myopic eye (simulation).

Figure 2 illustrates the transformation of images during precompensation. Retinal images (c and d) simulate the visual perception of a myopic observer looking at images a and b, respectively. The precompensated image (Figure 2b) itself appears excessively contrasty and generally strange, but becomes much closer to the original image after being blurred by the observer’s eye (Figure 2d).

The optimal precompensation varies for different refractive errors, so taking into account the specific refractive distortion introduced by a particular eye is commonly referred to as *personalization*. To formally describe the refractive error of the eye, it is customary to consider the eye as a lens system that forms an image on the retina, which is generally considered flat (since the angular size of the image of an object on the retina is usually small). Then, refractive error is characterized by the point spread function (PSF), which is a two-dimensional retinal image of a point object [7]. An eyeglass prescription or an aberrometric examination report (see, e.g., [8]) provides sufficient information to calculate the PSF of the human eye.

Precompensation can be used not only to compensate for visual acuity impairments, but also to correct aberrations introduced by VR headset lenses (e.g., Google Cardboard [9]) and to correct defocusing in projectors and other optical systems [10].

The main problem with precompensation is that it cannot be accurately implemented in practice: for an ideal image to form on the retina, some pixels in the precompensated image must have a brightness significantly exceeding the maximum possible for the display, while others must have a negative brightness, which is physically impossible. This problem is more pronounced with increasing the refractive error.

It is important to note that practically possible (but imprecise) solutions to the precompensation problem vary significantly in terms of clarity, contrast, and artifacts in the resulting retinal image. This, in turn, places high demands on the metrics by which various precompensation methods are compared: since a perfect solution is impossible, the best one must be chosen in accordance with the features of human perception and verified through human studies.

Existing precompensation algorithms employ one of two basic approaches [11]. The first approach decomposes the precompensation problem into deconvolution without restrictions on the permissible luminance and subsequent tone mapping. This allows the deconvolution problem to be solved analytically using Wiener filtering. However, such decomposition lacks convincing mathematical rationale and seems to be a lucky guess without investigated limits of applicability. The second approach directly solves the deconvolution problem with built-in restrictions, using numerical optimization. The use of deep learning, which approximates the second approach, is recognized as promising [6], but to date, no personalized neural network methods have been proposed for solving the precompensation problem.

The architectures suggested for the related non-blind deblurring problem for the first glance could provide the personalized solution, because they have two inputs (image and PSF). However, they do not take into account the limitations on the permissible luminance range and therefore cannot be applied directly. So, the relevant challenge is constructing a dual-input neural network architecture that generates a precompensated image with the limited luminance range typical of conventional displays and comparing its quality and performance with previously known methods. However, a systematic comparison of previously known methods has not yet been performed, so implementing this is also a pressing task.

The novelty of this paper lies in investigating the effectiveness of using dual-input neural networks for personalized precompensation of refractive errors of the human eye. Given the progress in related fields, the highest quality of solution can be achieved by a neural network approach; however, all previously proposed architectures did not allow personalization at the inference stage. This is precisely what makes the dual-input architecture interesting for investigation. The detailed contributions we claim are listed at the end of the Related works section, in order to more clearly demonstrate the distinctions from prior work.

## 2. Related Works

In this section, we consider previously known precompensation algorithms. The full story of their development can be found in a recent detailed review [11]. In this paper, we provide just a brief overview of these algorithms and summarize their features in appropriate tables.

The first works by Peli et al. on image precompensation appeared back in the 1980s [12,13] and aimed to increase not the image sharpness, but rather its local contrast, taking into account the contrast sensitivity functions (CSF) of the human eye, understood as the average for the target group. Thus, these methods were fundamentally non-personalized. It is worth mentioning that local contrast enhancement methods continue to develop today [14], but with no connection to precompensation: for example, for underwater images [15,16].

The problem of sharpness compensation taking into account the CSF of a particular eye was first explored in the work of Alonso and Barreto [17]. The authors employed a convolutional model of retinal image formation [7] (that became commonly used in subsequent precompensation studies):

(1) r(x,y)=t(x,y)∗h(x,y),

where r(x,y) is the retinal image, t(x,y) is the presented image, and h(x,y) is the PSF of the particular eye. Then, if the user is shown a specially precorrected image,

(2) $$p\left(x,y\right)=t\left(x,y\right)*h^{-1}\left(x,y\right),$$

then the user, with their imperfect eye, will see the original image. The authors consider this problem as, in some sense, equivalent to the inverse filtering/deconvolution problem.

In general, the inverse function $h^{-1}\left(x,y\right)$ does not exist, and finding a solution requires a regularization. For instance, it can be the common L2 regularization, which leads to the well-known Wiener filtering formula [18]:

(3) $$P\left(f_{x},f_{y}\right)=\left[\frac{1}{H(f_{x},f_{y})}\frac{|H\left(f_{x},f_{y}\right){|}^{2}}{|H\left(f_{x},f_{y}\right){|}^{2}+K}\right]T\left(f_{x},f_{y}\right),$$

where $P(f_{x},f_{y})$, $H(f_{x},f_{y})$, and $T(f_{x},f_{y})$ are the Fourier transforms of the functions p(x,y), h(x,y), and t(x,y), respectively, and *K* is the regularization constant.

**Figure 3:**
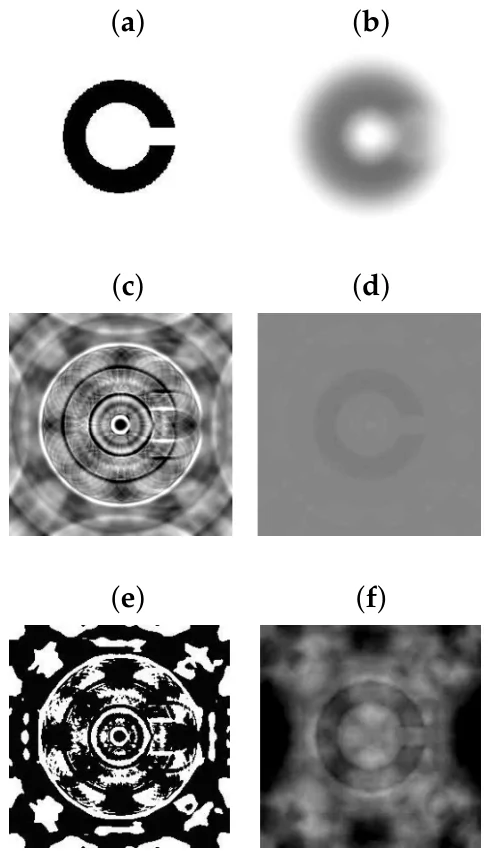
Contrast loss and precompensation artifacts caused by tone mapping: (**a**) original image; (**b**) corresponding retinal image (simulation); (**c**) precompensated image with linear tone mapping; (**d**) corresponding retinal image (simulation); (**e**) precompensated image with tone mapping that increases contrast in the middle of the dynamic range; (**f**) corresponding retinal image (simulation). The images used are taken from Alonso et al. [20].

In their papers published in 2005, Alonso et al. [19,20] first articulated the problem of limited available display luminance. Since deconvolution can produce an image that exceeds the actual luminance range of the image carrier (a monitor screen or the like), the authors propose applying tone mapping to it, bringing it within the available luminance range. However, tone mapping, which linearly reduces the dynamic range to the target, leads to an unacceptable loss of contrast, while attempts to increase it through nonlinear transformations lead to pronounced artifacts (see Figure 3). For instance, the histogram clipping proposed in the work of Huang et al. [21] yields (according to contrast-sensitive metrics) quality at the level of the Peli-Peli algorithm [22].

It should be noted that the quality of retinal images resulting from the tradeoff between high-contrast and artifact-free images can be partially improved using specialized multilayer displays with spatially separated layers [23]. Such methods, which require specialized, expensive hardware, are not considered in this paper. We focus on purely software-based methods applicable to any device having a display with controlled pixel brightness.

Ji et al. [24], in their human studies, demonstrated that people prefer high-contrast images with some artifacts over clear, low-contrast images without artifacts. They investigated the impact of ringing artifacts arising in precompensated images and, for the first time, proposed a personalized precompensation algorithm that produces sufficiently contrast retinal images. To achieve this, the tone mapping selection problem was posed and solved as an optimization problem, which, for the first time, allowed obtaining high-quality precompensated images.

Montalto et al. [25] proposed setting the optimization problem not as a post-processing (after the deconvolution), but as a deconvolution problem with constraints. To reduce the severity of artifacts, the authors used the total variation of the precompensated image as a regularizer:

(4) $$p_{m}=\underset{{\left||p|\right|}_{\infty }\leq 1}{\arg\;\min}\left(||h*p-{t||}_{2}+{\theta \;||\nabla p||}_{1}\right),$$

where $p_{m}$ is the resulting precompensated image, *h* is the eye’s PSF, *t* is the original (reference) image, and θ is the regularization coefficient. The authors, as in [24], controlled the tradeoff between artifact suppression and contrast enhancement in the perceived precompensated images using the parameter θ. Later, Güzel et al. [6] managed to improve the Montalto algorithm in terms of retinal image quality by abandoning the use of regularization while keeping the constraints.

It should be noted that current optimization approaches do not provide the performance required for real-time applications: they require several seconds per image on GPUs. Xu and Li [26] proposed an analytically computable modification of the Montalto method with the aim of improving its performance. However, the proposed analytical solution did not take into account the limitations on the range of acceptable luminances. The illustrations provided in the paper only allow comparison of the algorithm’s quality with the work of [27], which solves the non-blind deblurring problem without constraints. Although it is stated that the precompensation quality was compared with the Montalto algorithm, no quantitative metrics are provided in the paper. The source code for reproducing their algorithm has also not been published.

Güzel et al. [6] proposed accelerating their algorithm by approximating it with a neural network of the UNet architecture. For a single fixed PSF, this resulted in a performance increase of several orders of magnitude, but the authors did not propose a universal, personalized neural network algorithm.

Tanaka and Kawano [28] proposed a precompensation method based on a convolutional neural network of the VDSR (Very Deep Super Resolution) architecture. However, this method is not personalized, but rather tailored to a specific PSF.

This exhausts the list of algorithms solving the precompensation problem in its standard formulation. Several studies have proposed incorporating additional factors into the model of the human visual system. For example, Huang et al. [29] proposed dynamically accounting for changes in pupil size during observation. Ye et al. [30] proposed additionally accounting for the visual significance (saliency) of various image regions, prioritizing contrast preservation in the most significant areas while accepting lower quality in less important areas. Güzel et al. [6] proposed refining the eye model in terms of chromatic aberrations. As a result, the solution to the problem begins to depend, among other things, on the color primaries of the monitor used. Finally, Jiang et al. [31] proposed taking into account the dependence of the PSF on the user’s gaze direction for near-eye displays. These scenarios will not be considered in this paper, as they all require a real observer and cannot be processed in a single automatic benchmarking pipeline.

An overview of the considered precompensation algorithms can be found in Table 1. From these, we selected for comparison those personalized algorithms showing an acceptable level of quality, provided they are described in sufficient detail to be reproducible: namely, the algorithms by Ji, Montalto and optimization method by Güzel (Güzel-Opt, later referred to as Güzel).

Table 2 presents how the existing precompensation methods were benchmarked in known studies. In most cases, the authors compared their algorithm with few cases: either only with the no-precompensation case or with one or two competitors. Moreover, the comparison was often performed on single images or mini-datasets (not exceeding 10 images) for one or more PSFs.

When comparing different algorithms, either numerical metrics or human subjects (the authors themselves or invited test subjects) served as judges of the precompensation quality. These subjects were typically individuals with normal vision, who were shown physically-modeled or computer-simulated retinal images. The only attempt to test the quality of precompensation on people with refractive errors was reported by Xu and Li [26]. However, this paper does not provide characteristics of the subjects’ refractive errors, which significantly complicates the interpretation of the results. Modern studies benchmark not only the precompensation quality but also the speed of the algorithms.

So, this overview allows us to conclude that the existing precompensation methods face critical limitations. Classical optimization approaches like Güzel et al. [6] achieve reasonable quality but require several seconds per image on GPUs, making them impractical for real-time applications. Modern neural network methods, such as VDSR [28] and UNet [6], are not personalized: they require retraining for each user, which limits their practical deployment. Moreover, there is no systematic comparison of a wide range of precompensation algorithms on a single diverse dataset of images and PSFs modeling refractive errors of varying severity, across a wide range of metrics and human studies, as well as in terms of performance. All these problems will be addressed in the current paper.

The contribution of this paper is as follows:

- The use of dual-input neural networks for personalized precompensation of refractive errors of the human eye is proposed for the first time.
- An approach for adapting neural network models of non-blind deblurring for the problem of personalized precompensation is proposed and tested.
- A systematic comparison of state-of-the-art methods of personalized precompensation and new neural-network methods was conducted based on metrics and human studies.
- Thus, a new neural-network method of precompensation was constructed, superior in quality to previously known methods.

## 3. Materials and Methods

### 3.1. Datasets

**Figure 4:**
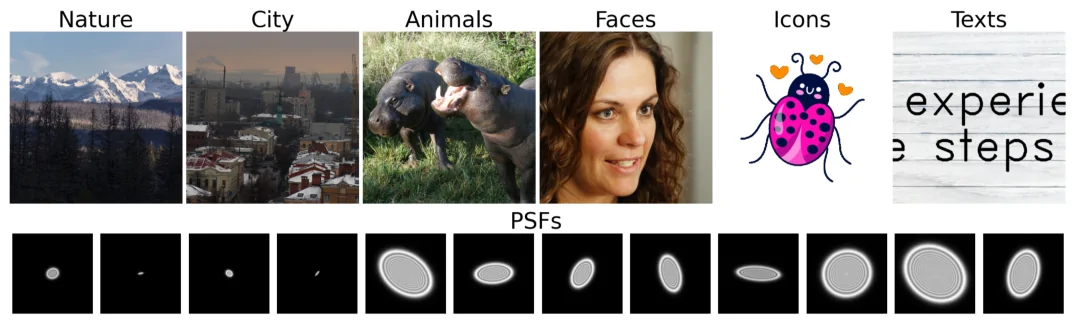
Sample data from the SCA-2023 dataset: (**top**) row—images by category; (**bottom**) row—examples of PSFs.

To solve the precompensation problem, besides the image to process, data on the PSF of the user’s eye are needed. Two datasets were used in our study: ImageNet-21k [32] and SCA-2023 [22]. The ImageNet-21k set was used exclusively for training neural networks (not used for testing). The SCA-2023 set contains examples of both presented images and PSFs (see Figure 4). Images from this set were used to test the models, while PSFs were used in both the training and testing phases.

**Figure 5:**
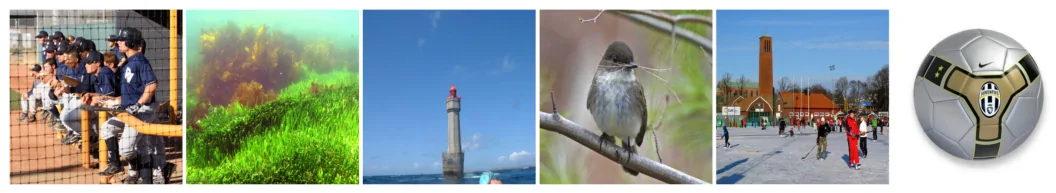
Randomly selected samples from the ImageNet-21K dataset (after resizing).

To train the selected models, 1000 images were randomly picked from the ImageNet-21k dataset, which satisfied the following conditions: the aspect ratio ranged from 1:1 to 4:3, and the minimum size of the smaller side was at least 512 pixels. All the images were then reduced to a fixed size of 512 × 512 pixels. For this purpose, each image was first isotropically (with preservation of aspect ratio) scaled so that the smaller side reached 512 pixels, and then a fragment of the required size was cut out from the central portion of the scaled image. The scaling was based on bilinear interpolation. The final result of resizing is shown in Figure 5.

As a source of PSFs, we used data from the SCA-2023 dataset. From it, all 768 available PSFs were selected, from which 384 samples were randomly picked for training. The eye pupil diameter (on which the PSF size also depends) was set at a typical value of 3.5 mm; and the angular width of the target image was set at 12°. Each of the 1000 preprocessed images was combined with each of the 384 selected PSFs to produce 384,000 unique dual-input sets for our neural network models. These data were divided into training (192,000 pairs) and validation (192,000 pairs) samples.

The SCA-2023 dataset containing 512×512 images of six different categories (e.g., portraits or text snippets) was used as a test dataset. All the 735 images were selected from it. The 384 PSFs that were not used to train the model were taken for testing. Thus, the test set included 735 images and 384 PSFs. From these, “image/PSF” pairs were formed by combining each image with each PSF, yielding 282,240 combinations. The resulting test dataset was subsequently used for both quantitative evaluation and human studies comparing different precompensation methods.

No additional data augmentation techniques were applied during the training. The training data were used in their original form after resizing and cropping. Cross-validation was not used; instead, a fixed train/validation/test split was employed throughout the study.

A fixed random seed was used for dataset splitting (12345) and the generation and randomisation of experimental pairs in the human studies (123). Random number generation was performed using the torch library.

### 3.2. Implementation of Classical Methods

The method of Montalto et al. [25] implies TV (total variation)-regularized numerical optimization of the discrepancy between the original (reference) image and the retinal projection of the precompensated image, calculated on the basis of the L2 metric, while ensuring that the pixel values of the precompensated image lie in the range [0,1]. The authors indicate that they used the FISTA (fast iterative shrinkage-thresholding algorithm) method [33] for the optimization, without going into implementation details. The original paper [25] neither references the source code nor specifies some of the parameters nor indicates whether FISTA/GP (gradient projection) or FISTA/FGP (fast gradient projection) was used.

In addition, Montalto et al. do not explicitly state whether an isotropic or anisotropic discretization of the total variation is used, while with the FISTA method [33] both discretizations can be used. Visual analysis of their illustrations suggests that Montalto et al. [25] used the simpler, anisotropic variation. This is confirmed by characteristic steplike artifacts, especially noticeable in homogeneous regions. Such artifacts are typical for anisotropic discretization, which independently considers horizontal and vertical derivatives, thus better preserving the orthotropic structure. To maximize the consistency with the original method, we implemented specifically the anisotropic version.

In our implementation, we used the FISTA/FGP version, as recommended by the authors of FISTA [33]. The main parameters of this method are the number of iterations, *N*, of the FGP algorithm; the regularization parameter, λ; and an estimate of the Lipschitz constant, *L*, of the target functional (The regularization parameter λ in the notation of FISTA [33] is related to the parameter θ from the expression (4) as θ=2λ). For the experiments, we empirically selected and used the following values of the above parameters: N=10, $\theta =2\;\lambda =10^{-6}$ and $L=5\times 10^{-2}$.

In the original paper by Ji et al. [24], the algorithm is not accompanied by the source code, and its parameters are also not given. We reproduced the algorithm from the methodological description provided by the authors. Since the implementation details were unavailable, the parameters were determined empirically by comparing the obtained results with the example images reported in the paper and selecting the values that provided the closest visual correspondence. The final parameter configuration used in our experiments was as follows:

- **Wiener filter:** the regularization constant was set at $K=10^{-2}$.
- **Tone mapping:** the Bézier curve parameter step was set at Δt=0.01.
- **Subtask optimization:** all the subtasks were solved using the Adam method [34] with a break parameter, $gap=10^{-5}$. In subtasks τ and $\tau_{-}$, the learning rate parameter was $lr_{\tau }=10^{-3}$, while in subtask *m*: $lr_{m}=0.5$.
- **External loop:** the number of iterations was set at 100, while the stopping criterion was $10^{-3}$.

The algorithm proposed by Güzel et al. [6] solves the precompensation problem by minimizing the L2 distance between the reference and the retinal image, while restricting the brightness of the precompensated image to the range [0,1]. The Adam method is used for optimization, and the constraint is implemented by the PGD (projected gradient descent) method [35]. The authors had provided the source code, which we used in our experiments.

All of the above implementations are available at the PyOLimp repository [36].

### 3.3. Dual-Input Neural Network Architectures

In the paper by Chaganova et al. [37] among all neural-network methods of non-blind deconvolution, three papers that define the current state of the art in this field were singled out:

- Zhang et al. [38] proposed the USRNet with an iterative deployment architecture based on HQS optimization, which is consistent with the optimization nature of the precompensation problem.
- Dong et al. [39] developed the DWDN, which uses the Wiener filter, a classic method used in earlier approaches to precompensation.
- KerUNC (Nan and Ji [40]) employs an optimization unrolling architecture with separate stages for image reconstruction, artifact suppression, and PSF error correction, enabling the model to account for degradation inaccuracies and improve robustness to blur kernel errors.

According to the literature, all the three considered neural-network models (USRNet, DWDN and KerUnc) can be used to solve the precompensation problem, because they have a proper dual-input interface and solve a similar problem. However, they need to be somehow adapted since the two problems are still different.

### 3.4. Adaptation of Dual-Input Neural Networks for the Precompensation Problem

**Figure 6:**
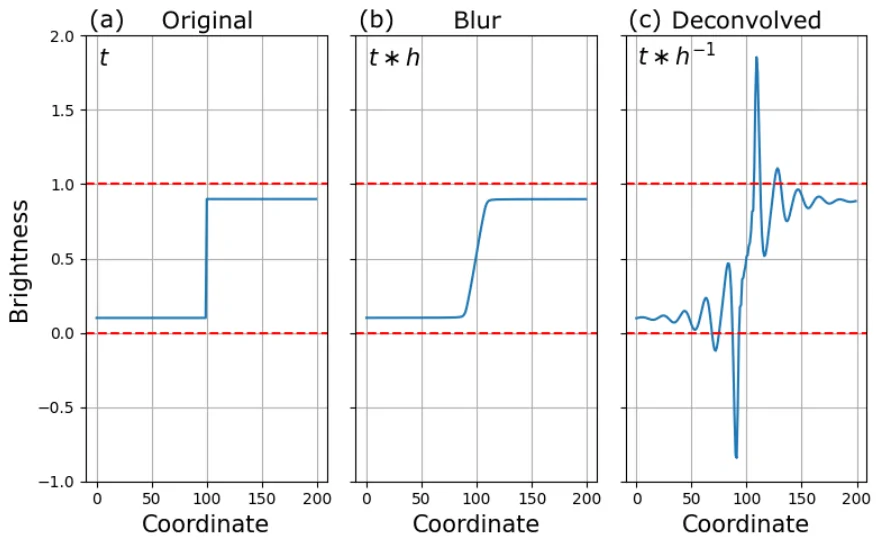
Out-of-range issue during precompensation: (**a**) the original signal *t*; (**b**) the perceived signal (the convolution t∗h of the original signal and the eye PSF); (**c**) the deconvolution $t*h^{-1}$ of the original signal. The red dashed lines denote the brightness range that can be displayed on the carrier and, consequently, used for precompensation.

What distinguishes the precompensation problem from non-blind deconvolution is that the deconvolved image as a mathematical function may have a very large range of brightnesses, which cannot be displayed on a physical carrier, such as a self-luminous display, paper, etc. Moreover, the deconvolved image may contain points with a negative brightness, which makes no sense in the case of precompensation. Consequently, the image formed as a result of the problem solution should be constrained within the allowable range of brightnesses. This is illustrated in Figure 6. For simplicity, we chose a one-dimensional signal: an image line with a contrasting step in brightness. It is clear from Figure 6c that the deconvolved image cannot be shown on any display.

As a rule, in the deconvolution problem, no restrictions are imposed on the output image. First, it is assumed that the reconstructed image existed in reality, and a close reconstruction of it will also turn out to be mappable. Second, the physical representability is not a fundamental requirement in the task of image reconstruction, since the full processing pipeline can be completed by recognizing the image rather than presenting it to a human.

In the course of adaptation of the neural network architectures selected in Section 3.3, the following changes were made to each of them in order to account for the specificity of the precompensation problem:

- A layer with the Sigmoid limiting function was added at the end of the network to ensure that the output values remain in the specified range [0, 1]:
  (5) $$Sigmoid\left(x\right)=\frac{1}{1+e^{-x}},$$
  where *x* is the vector of values taken from the previous layer.
- If the network had additional parameters besides the input image and PSF, they were fixed and were not changed during training or inference. For example, the DWDN and USRNet models have a scale parameter, and it was fixed at 1; the USRNet model also has a noise level parameter, and it was set to 0, because the original image is noise-free in the precompensation problem.

**Figure 7:**
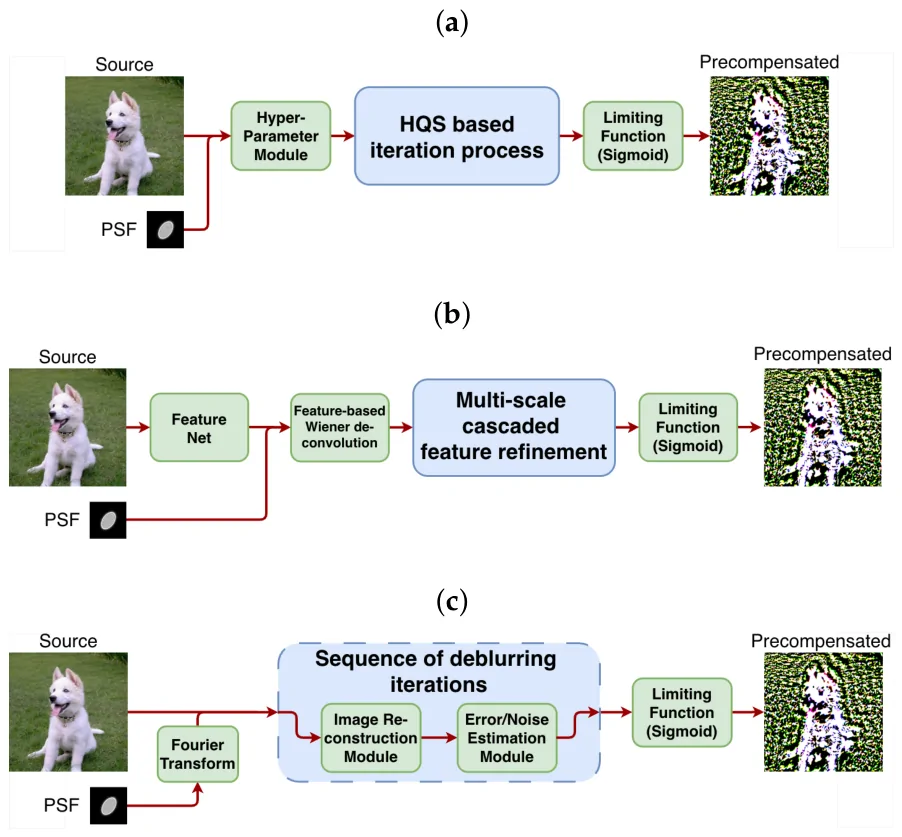
Interface diagrams of the neural networks modified for the precompensation problem: (**a**) USRNet-PC (here HQS stands for half-quadratic splitting), (**b**) DWDN-PC, and (**c**) KerUnc-PC.

As illustrated in Figure 7, the proposed adaptation of the three neural network architectures is rather a unified approach than individual transformation of each architecture. The adapted architectures are denoted hereafter by the postfix “-PC” (PreCompensation): USRNet-PC, DWDN-PC, and KerUnc-PC, respectively.

**Figure 8:**
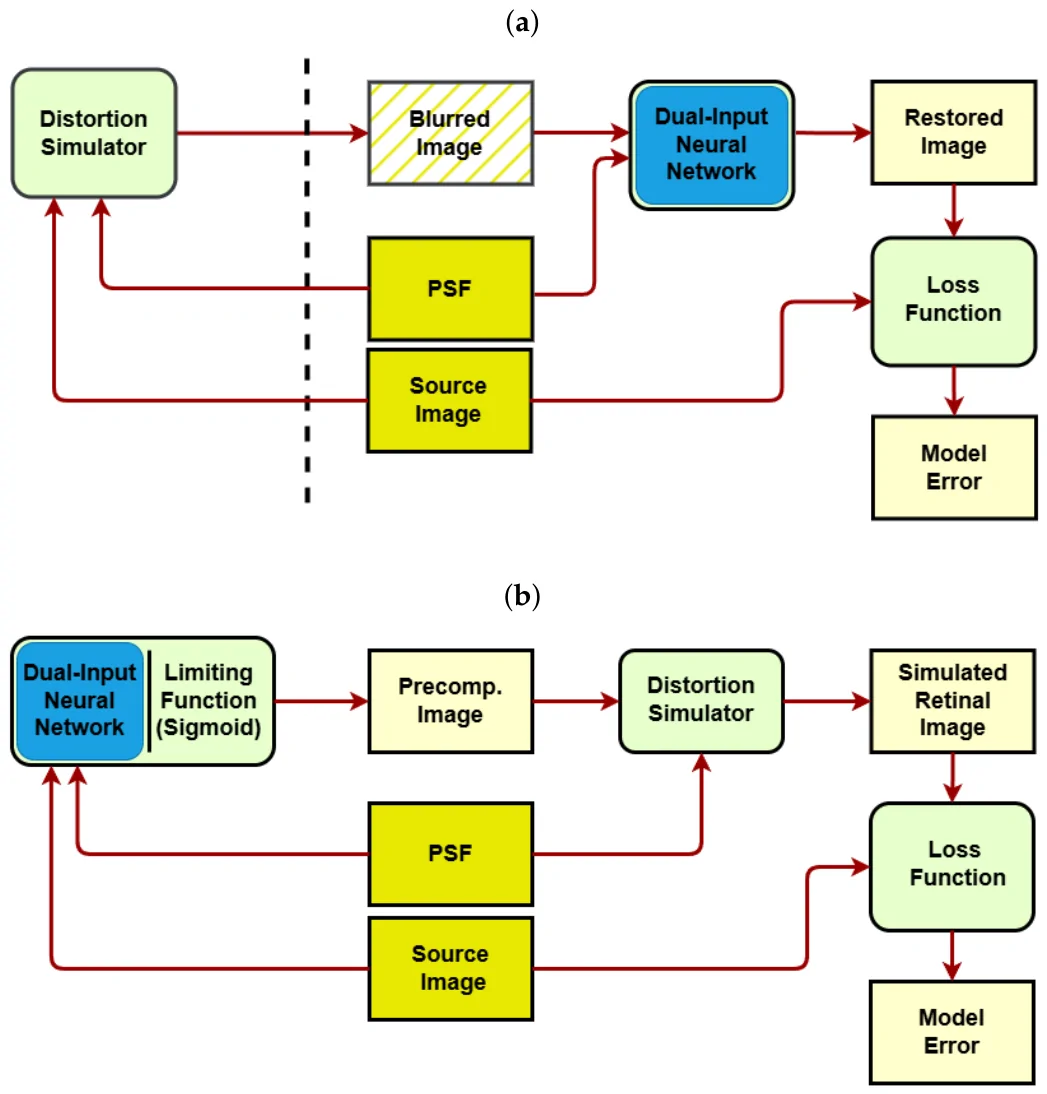
Data processing pipelines using dual-input neural networks: (**a**) for non-blind deconvolution; (**b**) for precompensation. Dark yellow blocks denote input data, light yellow—computable data. Green is for software modules, blue indicates the part of the neural network common to both schemes (**a**,**b**).

In addition, appropriate changes were made in the data processing pipeline used in model training and testing (see Figure 8). Speaking briefly, the dual-input neural network and the distortion simulator were swapped in accordance with the task change: in precompensation, the network is used to transform the original image so that the image at the output of the distortion simulator is close to the original one. This swap has two consequences. First, the distortion cannot be applied to the data in advance, but must be implemented at each training epoch. Second, the distortion simulation unit must allow for back error propagation, otherwise training becomes impossible with this scheme. This is not a problem since in our case the distortion is modeled by convolution (see Equation (1)), and modern deep learning frameworks, such as PyTorch and TensorFlow, provide differentiable implementations for it.

### 3.5. Loss Function

To select the loss function for training the neural network models, we preliminarily assessed their suitability using the following method. For each loss function, we performed optimization in the image space to find an image whose retinal projection is as close as possible to the target image, based on a given metric. Commonly used metrics, MS-SSIM and NRMSE, were considered, as well as CORR, which showed good agreement with human perception, according to [41].

**Figure 9:**
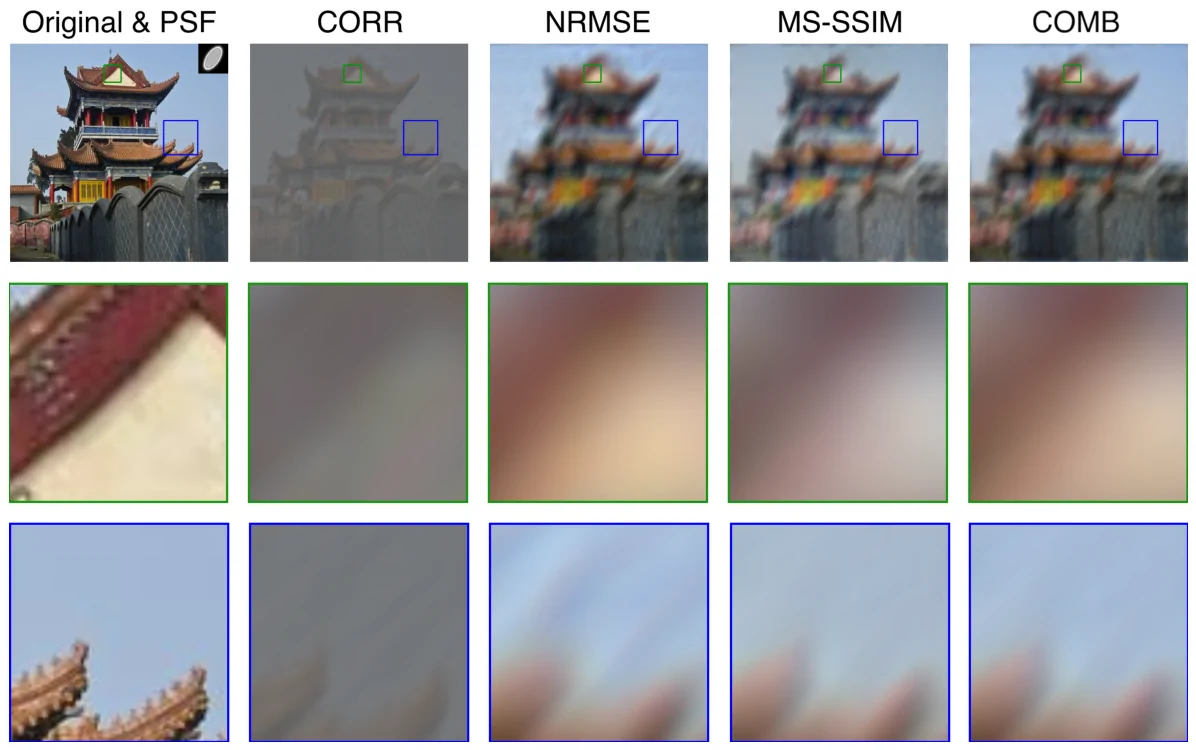
Comparison of retinal images obtained with different target functions for image precompensation. The second and the third rows show two magnified areas of the whole image given in the first row. The CORR function results in significant contrast loss, NRMSE introduces ringing artifacts, and MS-SSIM suppresses ringing at the expense of color reproduction. The combined target function COMB achieves a balance between artifact suppression, color preservation, and structural quality.

The results of this analysis are shown in Figure 9. As it can be seen, using CORR can lead to an unacceptable loss of contrast, MS-SSIM—to color distortion, and NRMSE—to artifacts. Considering this, we chose a weighted sum of MS-SSIM and NRMSE as a combined loss function:

(6) COMB(a,b)=0.5·NRMSE(a,b)+0.5·(1−MS-SSIM(a,b)),

where *a* and *b* denote the two images being compared by the loss function.

This loss function was used only for training our models. Its choice had no impact on the quality of the trained models or previously known algorithms. The success of this choice will become clear when discussing the results of human studies.

### 3.6. Training the Network Models

Training was performed using the PyOlimp framework [36] based on the PyTorch 2.7 library on two NVIDIA GeForce RTX 2080 Ti graphics cards according to scheme on Figure 8b. All the input images were normalized to bring the pixel values to the range [0, 1], for which each value was divided by 255. The PSFs were normalized by dividing their elements by the sum of all the values.

The loss function for the network *n*, the input image *t*, and the PSF *h* was defined as:

(7) Loss(n,t,h)=COMB(n(t,h)∗h,t).

The Adam optimizer [34] was used to optimize the neural network parameters. A training step of $1\times 10^{-5}$ was used. The batch size was set to 2. All the models were initialized using publicly available pre-trained weights provided by the respective authors for non-blind deblurring and subsequently fine-tuned for image precompensation.

The stopping criterion was the absence of a decrease in the value of the loss function on the validation set during two consecutive epochs, which indicated the emergence of overtraining. When this condition was met, the training process was terminated, and the checkpoint with the lowest validation loss was selected for subsequent testing. The USRNet-PC, DWDN-PC, and KerUnc-PC models were trained for a maximum of 173, 200, and 70 epochs, respectively. The checkpoints used for evaluation corresponded to epochs 171, 198, and 68, where the lowest validation loss was achieved.

### 3.7. Quantitative Evaluation of Precompensation Methods

The same normalizing procedures as those at the training stage were used for testing: each test image was normalized to the range [0,1] by dividing the pixel values by 255, and each PSF was normalized by dividing all its elements by their sum. To model retinal images and assess their quality, the output of the network was convolved with the corresponding PSF, after which the structural similarity between the resulting image and the original image was evaluated. Since there is no generally accepted single metric for evaluating image precompensation quality, we used a set of quantitative metrics: MS-SSIM, SSIM, STRESS, NRMSE, CORR, FSIM [42], and LPIPS [43].

In addition to the quality, the running time was also measured for all the methods, using the PyOLIMP framework [36]. The experiments were conducted on three hardware platforms: an NVIDIA GeForce GTX 1660 Ti Mobile GPU, an AMD Ryzen 7 4800H CPU, and an ARM Rockchip RK3566 processor with Cortex-A55 cores. These platforms were intentionally selected as consumer-grade embedded hardware rather than high-end server accelerators, since practical image precompensation systems are expected to operate under limited computational resources.

For runtime evaluation, we selected 10 image/PSF pairs from different categories of the test dataset. The same set of image/PSF pairs was used for all the methods and all the hardware platforms. For each method, the runtime was measured on each pair, and the final running time was obtained by averaging over these measurements. For the GPU experiments, the runtime was measured using CUDA events via torch.cuda.Event, which provides accurate timing of asynchronous GPU execution. For the CPU x86 and ARM experiments, the runtime was measured using the Python 3.13 high-resolution timer time.perf_counter.

### 3.8. Human Studies

Human studies were conducted on simulated retinal images with no personalized precompensation for each observer. For each randomly selected pair of the original image and the PSF from the dataset, precompensated images were generated using two randomly selected methods from among those being compared, after which the corresponding retinal images were obtained by convolving their results with the same PSF.

**Figure 10:**
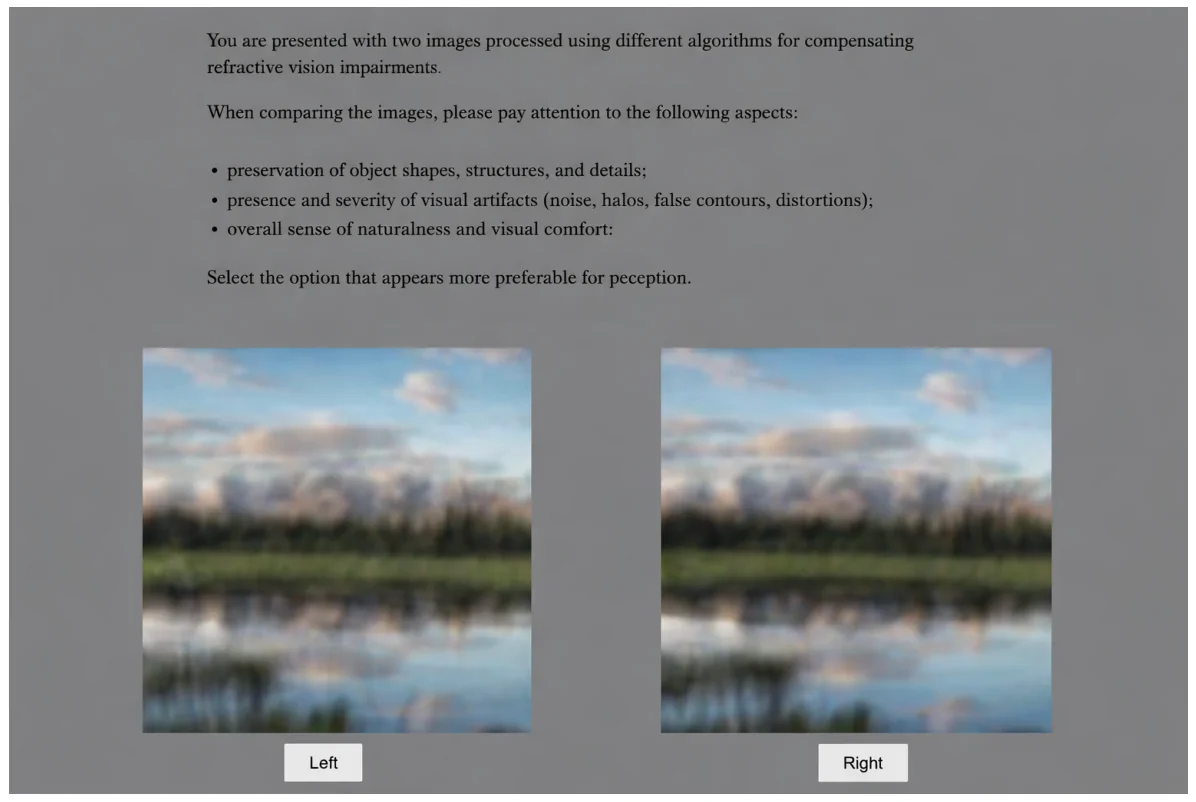
Sample page for the human studies.

The participants were shown pairs of retinal images, displayed on their monitor screen (see Figure 10). The position of each method (left or right) was selected randomly for each pair. The images were displayed on a 24-inch 16:9 monitor and occupied approximately 26% of the screen width, the viewing distance being 50 cm.

The participants were asked to choose the image seeming more preferable in terms of scene structure and visual integrity, according to the 2AFC (two-alternative forced choice) protocol [44,45]. When comparing the images, participants were advised to pay attention to the severity of visual artifacts, including noise, halos, false contours, and distortion of details.

The human study consisted of three stages. At the first stage, three previously known precompensation algorithms were compared: those by Montalto, Ji, and Güzel. At the second stage, three algorithms proposed by us were compared: USRNet-PC, DWDN-PC, and KerUnc-PC. At the third stage, the algorithm that demonstrated the best results among the previously known algorithms was compared with the best of the algorithms we proposed. The three-stage procedure allowed us to reduce the workload on the participants.

For statistical analysis, a mixed-effect logistic regression model was used, with participant identity included as a random effect to account for repeated responses from the same participant. The mean values, standard deviation, and confidence intervals for the regression parameters were calculated by the Bayesian model.

## 4. Results

### 4.1. Quantitative Evaluation

The quality of precompensation was evaluated using simulated retinal images, by comparing them with the original images from the test dataset. The test dataset consisted of 735 images and 384 PSFs, all from the SCA-2023 dataset, for a total of 282,240 image/PSF pairs.

We tested six different precompensation methods, including three SotA methods (by Montalto et al. [25], Ji et al. [24], and Güzel et al. [6]) and the three neural networks we adapted (USRNet-PC, DWDN-PC, and KerUnc-PC).

**Figure 11:**
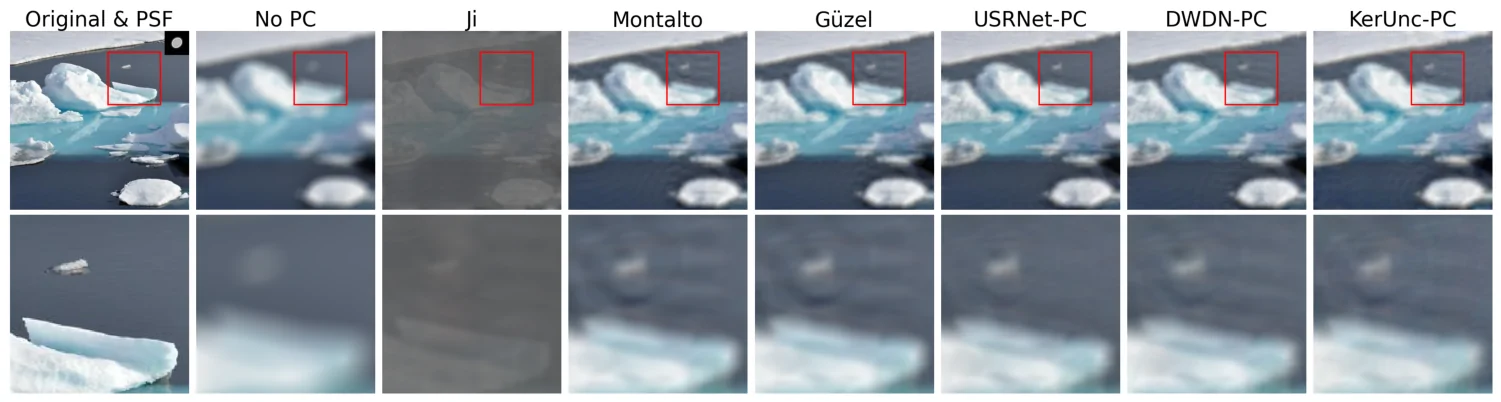
Example of how different precompensation algorithms process the same image picked from the Nature category. (**Top**) row: source image and PSF; simulated retinal images using different precompensation methods (the name of the method by the first author is indicated at the top). (**Bottom**) row: enlarged image fragment in red box.

**Figure 12:**
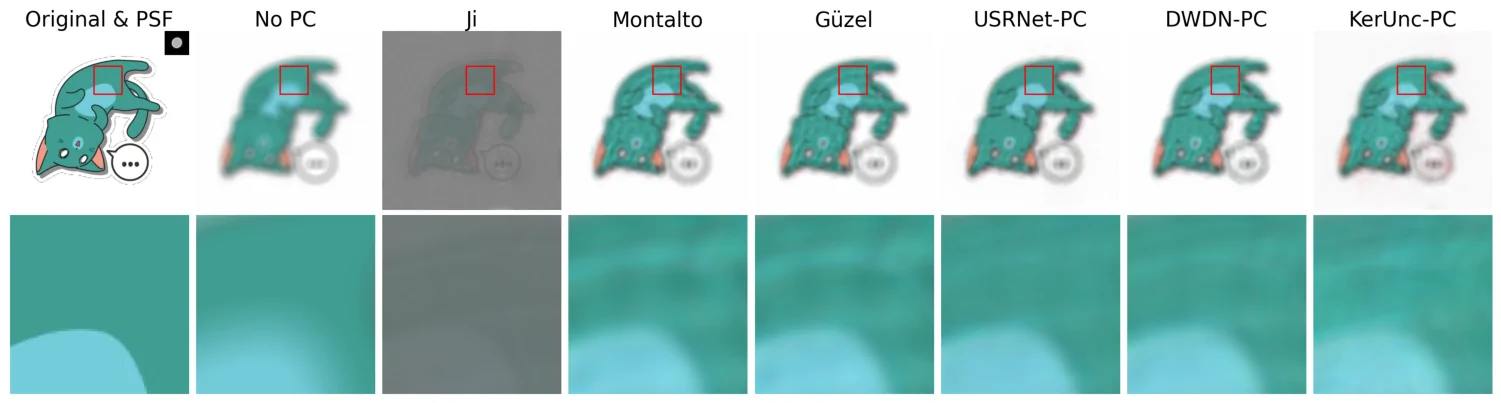
Example of how different precompensation algorithms process the same image picked from the Icons category. (**Top**) row: source image and PSF; simulated retinal images using different precompensation methods (the name of the method by the first author is indicated at the top). (**Bottom**) row: enlarged image fragment in red box.

**Figure 13:**
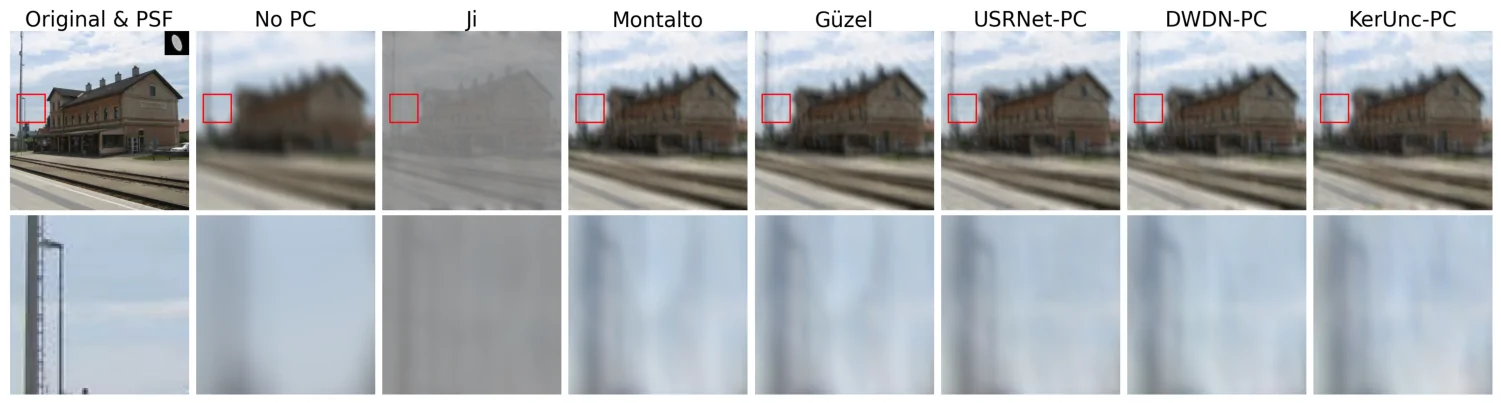
Example of how different precompensation algorithms process the same image from the City category. (**Top**) row: source image and PSF; simulated retinal images using different precompensation methods (the name of the method by the first author is indicated at the top). (**Bottom**) row: enlarged image fragment in red box.

The quality metric values are shown in Table 3, and examples of the results are shown in Figure 11, Figure 12 and Figure 13. The table shows that the Ji method performs worse than the other methods across the evaluated quality metrics and also performs worse than no precompensation. The results of the other methods are similar to one another, with different methods performing best on different metrics; however, all other methods are noticeably better than no precompensation. Later in this paper, a user study will be conducted to determine which of the methods provides the best results in terms of human perception.

To assess the computational performance of the methods across different deployment scenarios, we selected three hardware platforms representing distinct classes of computing devices. The NVIDIA GeForce GTX 1660 Ti Mobile represents a consumer-grade GPU accelerator, the AMD Ryzen 7 4800H represents a conventional high-performance x86 CPU platform, and the ARM-based Rockchip RK3566 represents low-power mobile and embedded devices.

The runtime comparison is presented in Table 4. The computational efficiency of the evaluated methods depends strongly on both the algorithmic approach and the hardware platform. USRNet-PC achieved the shortest inference time on the GPU, whereas DWDN-PC demonstrated the best performance on both x86 and ARM CPUs.

### 4.2. Human Studies

The human studies consisted of three stages. At the first stage, three previously known pre-compensation algorithms were compared: by Montalto, Ji, and Güzel. At the second stage, the three algorithms we proposed were compared: USRNet-PC, DWDN-PC, and KerUnc-PC. At the third stage, the algorithm that showed the best results among the previously known ones was compared with the best of the algorithms we proposed.

The study was conducted entirely online and anonymously. Therefore, participants’ age, sex, visual status, and the use of optical correction (glasses or contact lenses) were not collected. No specific inclusion or exclusion criteria were applied, and the visual acuity and color vision were not assessed separately. However, the participants were instructed to take part only if they had normal vision or vision corrected to normal using glasses or contact lenses, and to use their usual optical correction during the experiment. Due to the anonymous nature of the study, participant identities were not tracked across the three stages; therefore, some participants might have taken part in more than one stage. Since the experiment was conducted remotely using the participants’ own devices, the displays were not calibrated, and the brightness, contrast, and ambient lighting conditions were not controlled. The only controlled display-related parameter was the screen size: the participants were required to use displays with a diagonal size between 23 and 27 inches.

For all the three stages, the image/PSF pairs presented to the participants were randomly sampled from the test dataset described in Section 3.1, which contained images from all the six categories and the PSFs reserved for testing.

At the first two stages of the experiment, 19 and 27 observers participated, comparing 460 and 673 pairs of images, respectively. Each participant was asked to compare 25 pairs of images, each having a resolution of 512×512 pixels (Figure 10). The outcome of the human studies was aggregation of the participants’ responses across all the presented image pairs. The summary distribution of participant choices is given in Table 5 and Table 6. Among the previously known algorithms, the Güzel method showed the best results, and among those we proposed, the USRNet-PC architecture performed best.

At the third stage of the studies, these two algorithms were compared to one another in order to figure out the final winner.

Table 7 and Table 8 provide detailed pairwise preference counts. Each off-diagonal cell indicates the number of times the method in the corresponding row was preferred over the method in the corresponding column. Thus, at the first stage, Güzel and Montalto were compared 171 times, Güzel and Ji 144 times, and Montalto and Ji 145 times. At the second stage, USRNet-PC and DWDN-PC were compared 228 times, USRNet-PC and KerUnc-PC 225 times, and DWDN-PC and KerUnc-PC 220 times.

At the third stage of the studies, 80 observers compared 1990 pairs of images (Table 9). The purpose of the statistical analysis was to determine whether the observed preference for USRNet-PC was a systematic rather than a random deviation from the equal probability of choosing the two methods. Since each observer performed several comparisons (21–25), their responses are not independent. Therefore, a mixed effects logistic regression (MELR) [46] was used, allowing for repeated responses using an individual random effect. We first consider a model with a fixed intercept $\beta_{0}$ and a random effect for each user *i*:

(8) $$\ln\left(\frac{p_{i}}{1-p_{i}}\right)=\beta_{0}+u_{i},$$

where $p_{i}$ is the probability that user *i* will pick the USRNet-PC method in comparison with Güzel. Random i.i.d. variables $u_{i}∼N\left(0,\sigma^{2}\right)$ represent individual deviations from the common trend $\beta_{0}$. The model parameters were estimated using variational Bayesian inference [47], which provides an approximation to the posterior distributions.

Based on the basic model (Table 10), we can conclude that USRNet-PC is significantly preferable to Güzel. Fixed intercept estimation $\beta_{0}=0.26$ corresponds to the probability of choosing a USRNet-PC: $P=1/(1+e^{-\beta_{0}})\approx 0.56$. After accounting for random effects, the effect remains stable. The variance for users is significantly greater than zero. This means that different people have different inclinations to choose USRNet (this is expected due to individual perception characteristics).

We also conducted a study on the effects of image content and PSF characteristics on user preferences. The images from the SCA-2023 dataset belong to six categories, which can be grouped into two visually distinct classes: images containing real objects (Faces, Nature, Animals, Urban), for which users are more sensitive to naturalness and detail preservation, and images containing GUI elements (Texts, Icons), for which readability and clean, sharp edges are particularly important. To investigate the preference for each group, we added a categorical variable *t*, which is equal to 0 if the image belongs to the first group, and 1 otherwise. Each PSF in this dataset is described by three parameters (sphere *S*, cylinder *C*, and angle *A*), which we transformed as follows: the sphere and cylinder were centered by subtracting their means over dataset, and the angle was converted to its sine. Thus, we used:

(9) $$s=S-\bar{S},\;c=C-\bar{C},\;a=\sin\left(\pi A/180^{\circ }\right).$$

Putting everything together, we formulated a more complex model to test the effect of the source images/PSFs on user preference:

(10) $$\ln\left(\frac{p_{ij}}{1-p_{ij}}\right)=\beta_{0}+\beta_{t}\cdot t_{j}+\beta_{S}\cdot s_{j}+\beta_{C}\cdot c_{j}+\beta_{A}\cdot a_{j}+u_{i},$$

where $p_{ij}$ is the probability that user *i* will pick the USRNet-PC method over Güzel in the *j*-th data sample; the parameter $\beta_{t}$ represents the image type variable $t_{j}$; and $\beta_{S},\beta_{C},\beta_{A}$ represent the processed PSF variables $s_{j},c_{j},a_{j}$, respectively. This parameters for fixed effects were estimated using the same Bayesian inference procedure as in the basic model. The structure of random effects remained the same.

The extended model for image categories and continuous PSF characteristics allowed us to clarify the conditions under which USRNet-PC is superior to Guzel (Table 11). The results show that:

- The image category is critically important. When the PSF parameters are set to their mean values, USRNet-PC is preferred over Güzel for general images (probability 52.7%), but its advantage is considerably stronger for icons and texts (probability 60.8%).
- The PSF parameters do not have a significant effect on USRNet-PC preference.
- Individual differences remain substantial, as the variance of the random intercept is still significantly different from zero.

Taken together, our mixed effects logistic regression analysis demonstrates that USRNet-PC is, on average, preferred over Güzel, but this preference is highly dependent on the image type. The advantage is most pronounced for images containing text or icons, while for the other categories (animals, natural scenes, urban scenes, and faces) the preference is weaker: only slightly above chance. The PSF parameters (sphere, cylinder, and angle) do not appear to modulate this effect significantly in our data. The strong user-specific random intercept indicates that individual viewing conditions or perceptual strategies may play a role, but even after accounting for this heterogeneity, the image-category effect remains robust.

Sample images precompensated using different methods are shown in Figure 11, Figure 12 and Figure 13. In these images, all of the methods under consideration, with the exception of the Ji method, provide similar sharpness and contrast; however, only the images obtained using the USRNet method are almost free of artifacts (see the enlarged areas).

## 5. Discussion

Thus, we have shown that neural network architectures designed for deblurring, but adapted to precompensation, can produce results of superior quality to previously known precompensation algorithms. If we were to propose a new, previously unknown neural network architecture, it would require an ablation study. However, our approach is based on previously published architectures, so analyzing the influence of the individual components of which they are composed is not the goal of our research.

**Figure 14:**
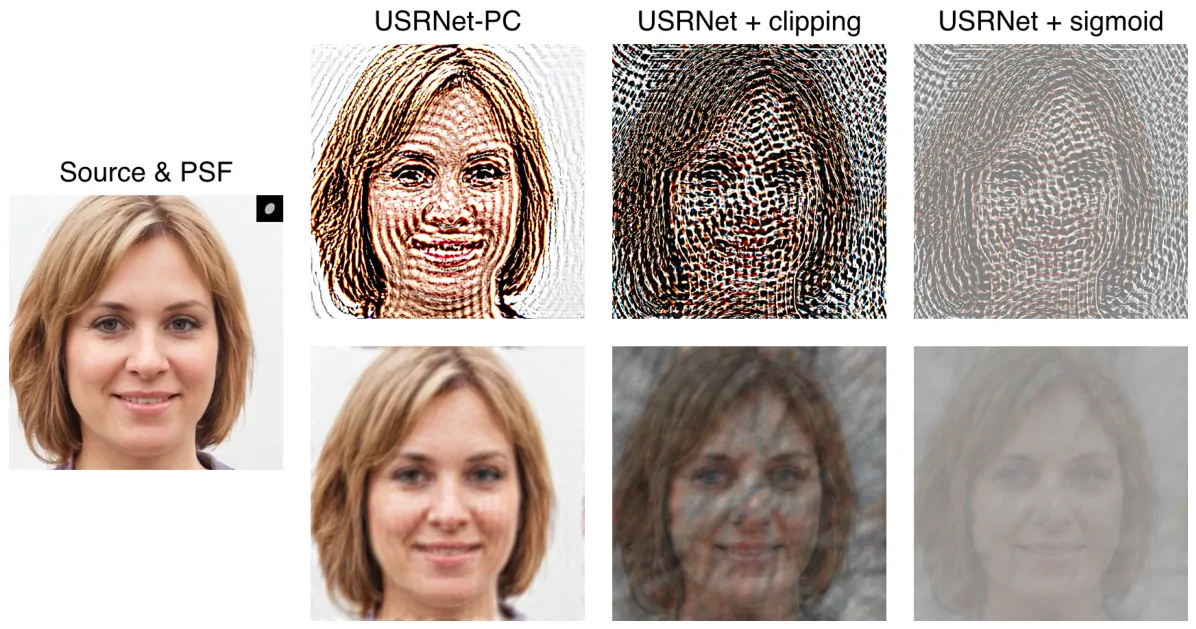
Comparison of the adapted neural network model, USRNet-PC, with non-adapted USRNet, equipped with different post-processing modules. (**Left**) column: source image and PSF. (**Top**) row: precompensated images obtained with each of the three models. (**Bottom**) row: corresponding retinal images.

Nevertheless, we have checked the point that is specifically relevant to our case: that limiting the neural network output values to the interval [0,1] is required not only during neural-network model inference but also during its training. Figure 14 presents the results of precompensation using the classic USRNet model with two post-processing options (clipping and sigmoid) and the USRNet-PC model (which we obtained through adaptation). As can be seen, the post-processing falls short of achieving acceptable precompensation quality.

The differences between the results of quantitative benchmarking and human studies deserve a special discussion. According to the calculated metrics, most of the methods under consideration demonstrate similar results, with different metrics identifying different leaders. However, the user studies revealed a statistically significant preference for the USRNet-PC method.

This indicates that existing image quality metrics do not fully capture the peculiarities of human perception in the precompensation problem. It is important, as precompensation is not aimed at restoring the image as such, but at improving its human visual perception. Consequently, even small differences in artifact levels, local contrast, and the naturalness of object boundaries can have a significant impact on subjective quality assessments while remaining virtually imperceptible in terms of traditional metrics. This confirms the need for a human study as a mandatory complement to objective metrics when evaluating precompensation methods.

An important limitation of our human study is that the participants had normal vision and evaluated computer-simulated retinal images. Therefore, the study assesses perceptual preferences between simulated precompensation results rather than actual improvements in visual perception in users with myopia, hyperopia, or astigmatism. Evaluation with the intended user population remains an important direction for future work.

Although neural network models have shown higher quality and better performance, compared to previously known methods, an obvious risk of their use should also be noted. Training neural networks requires extensive datasets with a variety of PSFs. We have used the SCA-2023 [22] dataset, which covers a variety of PSFs and different image categories, but it is relatively small by the standards of classical machine learning tasks. Related questions about the generalization ability of the tested neural network methods also remain open. In particular, how effective they are when used with PSFs with characteristic widths different from those found in the training dataset.

In the review of neural-network methods of non-blind deconvolution [37] it is noted that the KerUnc and DWDN models show significant (including that with respect to the Wiener filter) robustness to errors in the PSF estimation. Such robustness is highly desirable for the precompensation problem, as it would allow one to level out variations in the distance between the eye and the screen, as well as variations in the eye pupil diameter. But, unfortunately, there is no sufficient justification to believe that the behavior of the models will be similar in the two problems under consideration.

## 6. Conclusions

In this paper, we propose the use of dual-input neural networks for personalized image precompensation. While the first input is the image to be precompensated, the second is for image blur parameters, specified as the point spread function of the observer’s eye. Using an original approach, we were the first to adapt three significantly different neural-network architectures designed for non-blind deblurring to the precompensation problem and conduct their systematic benchmarking against the existing solutions. The key feature of this benchmarking was the specially organized human study, in which, as is common in works of predecessors, the subjects were individuals with normal vision, who were shown computer-simulated retinal images, and therefore the study evaluates perceptual preferences under simulated conditions rather than clinical effectiveness in users with refractive errors. According to these human studies, one of the adapted architectures (USRNet-PC) demonstrates superior quality, outperforming state-of-the-art algorithms.

The main limitation of personalized precompensation methods is their low computational efficiency: both previously known algorithms and the proposed models are computationally complex and unsuitable for real-time operation. However, the fact that USRNet-PC was the fastest (among those compared) on a graphics accelerator (see Table 4 for more details), demonstrating a runtime more than 4.5 times shorter than the state-of-the-art approach by Güzel et al., while DWDN-PC was the fastest on a low-power central processor, demonstrating a runtime 7 times shorter than that of Güzel et al., suggests the potential for further development of the neural network-based approach.

## Figures and Tables

**Table 1:** Comparison of precompensation methods.

Method	Year	Type	Personalized	Color	Quality	Fully Described
Peli-Peli [13]	1984	Analytic	No	No	Low clarity	Yes
Alonso [19,20]	2005	Analytic	Yes	No	Low contrast	Yes
Huang [21]	2012	Analytic	Yes	No	Low contrast	Yes
**Ji** [24]	2014	Optimization	Yes	Yes	Acceptable	Yes
**Montalto** [25]	2015	Optimization	Yes	Yes	Acceptable	Yes
Xu-Li [26]	2018	Analytic	Yes	Yes	Acceptable	No
Tanaka [28]	2021	Neural Network	No	Yes	Acceptable	Yes
**Güzel-Opt** [6]	2023	Optimization	Yes	Yes	Acceptable	Yes
Güzel-Unet [6]	2023	Neural Network	No	Yes	Acceptable	Yes
**Our proposal**	2026	Neural Network	Yes	Yes	Acceptable	Yes

**Table 2:** Features of precompensation methods benchmarking.

Study	Year	Number of Cases Compared	Blur Method	Judges	Images	PSFs	Speed Comparison
Alonso [19]	2005	2	physical modeling	subjects	single	single	no
Huang [21]	2012	3	physical modeling	authors	single	single	no
Montalto [25]	2015	4	physical modeling	subjects	mini-dataset	mini-dataset	yes
Xu-Li (images) [26]	2018	2	real human eye	subjects	mini-dataset	mini-dataset	yes
Xu-Li (video) [26]	2018	2	computer simulation	authors	single video	not specified	yes
Tanaka [28]	2021	2	computer simulation	metrics	dataset	mini-dataset	no
Güzel [6]	2023	3	computer simulation	authors	single	single	yes
Alkzir [22]	2023	4	computer simulation	metrics	dataset	dataset	no
Our proposal	2026	7	computer simulation	metrics, subjects	dataset	dataset	yes

**Table 3:** Quality assessment of different image precompensation methods, using image similarity metrics. The “No PC” method means no precompensation at all. The names of the non-neural-network methods are given by the first author.

Metric	No PC	Montalto	Ji	Güzel	USRNet-PC	DWDN-PC	KerUnc-PC
MS-SSIM	0.6065	0.7262	0.4293	0.7302	**0.7478**	0.7387	0.7378
SSIM	0.5345	0.6371	0.4614	0.6422	**0.6586**	0.6523	0.6507
1-STRESS	0.8282	0.8518	0.6974	**0.8537**	0.8497	0.8481	0.8437
1-NRMSE	0.8244	0.8485	0.4760	**0.8505**	0.8452	0.8451	0.8252
CORR	0.8095	**0.8522**	0.8468	0.8515	0.8499	0.8465	0.8439
FSIM	0.6549	0.7368	0.6062	0.7369	0.7357	**0.7414**	0.7354
1-LPIPS	0.4225	0.4935	0.2989	0.5044	**0.5058**	0.5034	0.4720

**Table 4:** Comparison of the inference times of different precompensation methods. The names of the non-neural-network methods are given by the first author.

Method	GPU ^1^, s	CPU ^2^, s	CPU (arm) ^3^, s
USRNet-PC	**0.975**	20.243	531.005
DWDN-PC	1.591	**5.644**	**118.331**
KerUnc-PC	2.488	50.326	641.337
Güzel	4.388	21.778	843.736
Ji	2.537	7.197	464.432
Montalto	1.761	9.903	188.137

^1^ GPU NVIDIA GeForce GTX 1660 Ti Mobile; ^2^ CPU x86 AMD Ryzen 7 4800H; ^3^ ARM Rockchip RK3566 (Cortex-A55).

**Table 5:** Results of user comparison of the previously known methods.

Method	Number of Preferences	Preference Share (%)
Güzel	192	41.7%
Montalto	181	39.3%
Ji	87	18.9%
Total	460	100%

**Table 6:** Results of user comparison of the methods proposed by us.

Method	Number of Preferences	Preference Share (%)
USRNet-PC	303	45.0%
DWDN-PC	255	37.9%
KerUnc-PC	115	17.1%
Total	673	100%

**Table 7:** Detailed information about user preferences in previously known methods.

		Alternative		
		Güzel	Montalto	Ji
Picked	Güzel	—	87	105
	Montalto	84	—	97
	Ji	39	48	—

**Table 8:** Detailed information about user preferences in methods proposed by us.

		Alternative		
		USRNet-PC	DWDN-PC	KerUnc-PC
Picked	USRNet-PC	—	130	173
	DWDN-PC	98	—	157
	KerUnc-PC	52	63	—

**Table 9:** Results of user comparison of the winners of Table 5 and Table 6.

Method	Number of Preferences	Preference Share (%)
USRNet-PC	1120	56.3%
Güzel	870	43.7%
Total	1990	100%

**Table 10:** Basic MELR results. Parameter types are mean structure (M) and variance structure (V). The significance of the coefficient is determined by whether its 95% creditable interval contains zero.

Parameter	Type	Posterior Mean	Posterior SD	Significance
β	M	0.26	0.05	Yes
σ	V	0.23	0.02	Yes

**Table 11:** Complex MELR results. Parameter types are mean structure (M) and variance structure (V). The significance of the coefficient is determined by whether its 95% creditable interval contains zero.

Parameter	Type	Posterior Mean	Posterior SD	Significance
$\beta_{0}$	M	0.10	0.05	Yes
$\beta_{t}$	M	0.34	0.07	Yes
$\beta_{S}$	M	−0.03	0.02	No
$\beta_{C}$	M	−0.04	0.03	No
$\beta_{A}$	M	−0.04	0.06	No
σ	V	0.23	0.02	Yes

## Data Availability

The training and test datasets are publicly available on Zenodo (https://zenodo.org/records/15926161, accessed on 16 September 2026). The source code is available at GitHub (https://github.com/pyolimp/pyolimp/tree/e3db3bf2897753aa8d27ec8b299621ee6a77dd35, accessed on 16 September 2026) (revision e3db3bf2897753aa 8d27ec8b299621ee6a77dd35), and the pretrained weights for all evaluated architectures are available at Hugging Face (https://huggingface.co/pyolimp/pyolimp/tree/main/RVI, accessed on 16 September 2026) (revision 3699d7c, DOI: https://doi.org/10.57967/hf/4235, accessed on 16 September 2026).

## Abbreviations

The following abbreviations are used in this manuscript:

2AFC	Two-Alternative Forced Choice
CORR	Correlation coefficient
DWDN	Deep Wiener Deconvolution Network
FGP	Fast Gradient Projection
FISTA	Fast Iterative Shrinkage-Thresholding Algorithm
FSIM	Feature Similarity Index Measure
GP	Gradient Projection
HQS	Half-Quadratic Splitting
HVS	Human Vision System
KerUnc	Kernel Uncertainty
LPIPS	Learned Perceptual Image Patch Similarity
MELR	Mixed Effects Logistic Regression
MS-SSIM	Multi-Scale Structural Similarity Index Measure
NRMSE	Normalized Root Mean Square Error
PC	PreCompensation
PSF	Point Spread Function
SCA	Sphere, Cylinder, Axis
SD	Standard Deviation
SSIM	Structural Similarity Index Measure
STRESS	STandardised REsidual Sum of Squares
USRNet	Unfolding Super-Resolution Network
VDSR	Very Deep Super-Resolution
VR	Virtual Reality
